# Supplementary material for: Disruption of FOXO3a-miRNA feedback inhibition of IGF2/IGF-1R/IRS1 signaling confers Herceptin resistance in HER2-positive breast cancer
Source: Nat Commun. 2021 May 11;12:2699. doi: 10.1038/s41467-021-23052-9 (PMC8113606; doi:10.1038/s41467-021-23052-9)
Supplement: Supplementary file 1 — Supplementary Information [file 41467_2021_23052_MOESM1_ESM.pdf]

Supplementary Information

Disruption of FOXO3a-miRNA feedback inhibition of IGF2/IGF-1R/IRS1 signaling confers Herceptin resistance in HER2-positive breast cancer

Liyun Luo[1#], Zhijie Zhang[1#], Ni Qiu[1#], Li Ling[1], Xiaoting Jia[1], Ying Song[1], Hongsheng Li[1], Jiansheng Li[1], Hui Lyu[2], Hao Liu[1], Zhimin He[1*], Bolin Liu[2*], and Guopei Zheng[1,3*]

[1]Affiliated Cancer Hospital & Institute of Guangzhou Medical University, Guangzhou, Guangdong 510095 China

[2]Department of Genetics, Stanley S. Scott Cancer Center, School of Medicine, Louisiana State University (LSU) Health Sciences Center, New Orleans, LA 70112 USA

[3]Guangzhou Municipal and Guangdong Provincial Key Laboratory of Protein Modification and Degradation, Guangzhou, Guangdong 510095 China

[#] These authors contributed equally: Liyun Luo, Zhijie Zhang, Ni Qiu

Supplementary figures

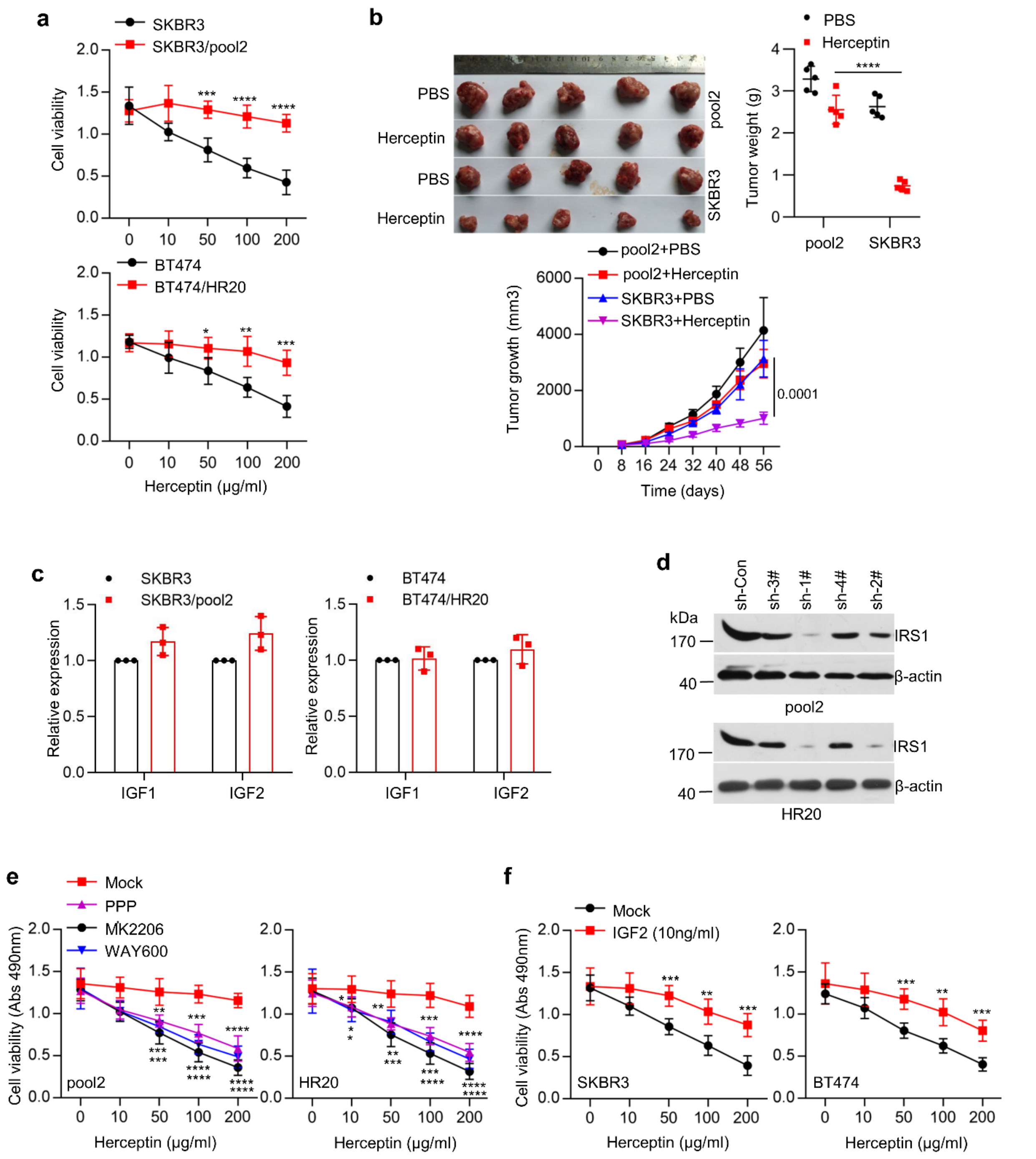

Supplementary figure 1. IGF2/IGF-1R/IRS1/Akt/mTOR signaling pathway was involved in resistance to Herceptin in HER2-positive breast cancer cells.

a SKBR3, SKBR3/pool2 (pool2), BT474, or BT474/HR20 (HR20) cells were treated with Herceptin at indicated concentrations for 72 hrs. Cell viability was evaluated by MTS assays, SKBR3/pool2: *** p=0.0003, **** p<0.0001, BT474/HR20: * p=0.0184, ** p=0.002, *** p=0.0004. **b** SKBR3 or pool2 cells were inoculated subcutaneously into the armpit of female Balb/C athymic nude mice to

generate tumor xenografts. When the tumor size reached ~100 mm^3, the mice were randomly grouped and injected intraperitoneally (i.p.) with PBS or Herceptin (10 mg/kg) (n=5) once every 5 days. At the experimental endpoint, tumors were excised and pictured. The tumor weights and sizes were also measured, **** $p<0.0001$. **c** The mRNA expression levels of *IGF1* and *IGF2* in the cells were measured by qRT-PCR. **d** Pool2 or HR20 cells were transfected with control shRNA (sh-Con) or specific *IRS1* shRNAs (sh-1#, sh-2#, sh-3#, or sh-4#). The expression of IRS1 was examined by western blot assays, Data show a representative of three independent experiments. **e** Pool2 or HR20 cells were treated with vehicle (Mock) or PPP (1 μM), MK2206 (1 μM), or WAY600 (1 μM) in combination with Herceptin at indicated concentrations for 72 hrs. Cell viability was evaluated by MTS assays, Pool2 PPP: ** $p=0.0022$, *** $p=0.0001$, **** $p<0.0001$, Pool2 MK2206: *** $p=0.0008$, **** $p<0.0001$, Pool2 WAY600: *** $p=0.0007$, **** $p<0.0001$, HR20 PPP: * $p=0.0456$, ** $p=0.0018$, *** $p=0.0003$, **** $p<0.0001$, HR20 MK2206: * $p=0.0312$, *** $p=0.0009$, **** $p<0.0001$, HR20 WAY600: * $p=0.0298$, ** $p=0.0076$, *** $p=0.0001$, **** $p<0.0001$. **f** SKBR3 or BT474 cells were treated with rhIGF2 (10 ng/ml) in combination with Herceptin at indicated concentrations for 72 hrs. Cell viability was evaluated by MTS assays, SKBR3: *** $p=0.0007$, ** $p=0.0015$, *** $p=0.0003$, BT474: *** $p=0.0006$, ** $p=0.0011$, *** $p=0.0003$. Statistical significance was determined by a two-tailed Student's t test (a, b, e, f). Data show a representative of three independent experiments. Source data are provided as a Source Data file.

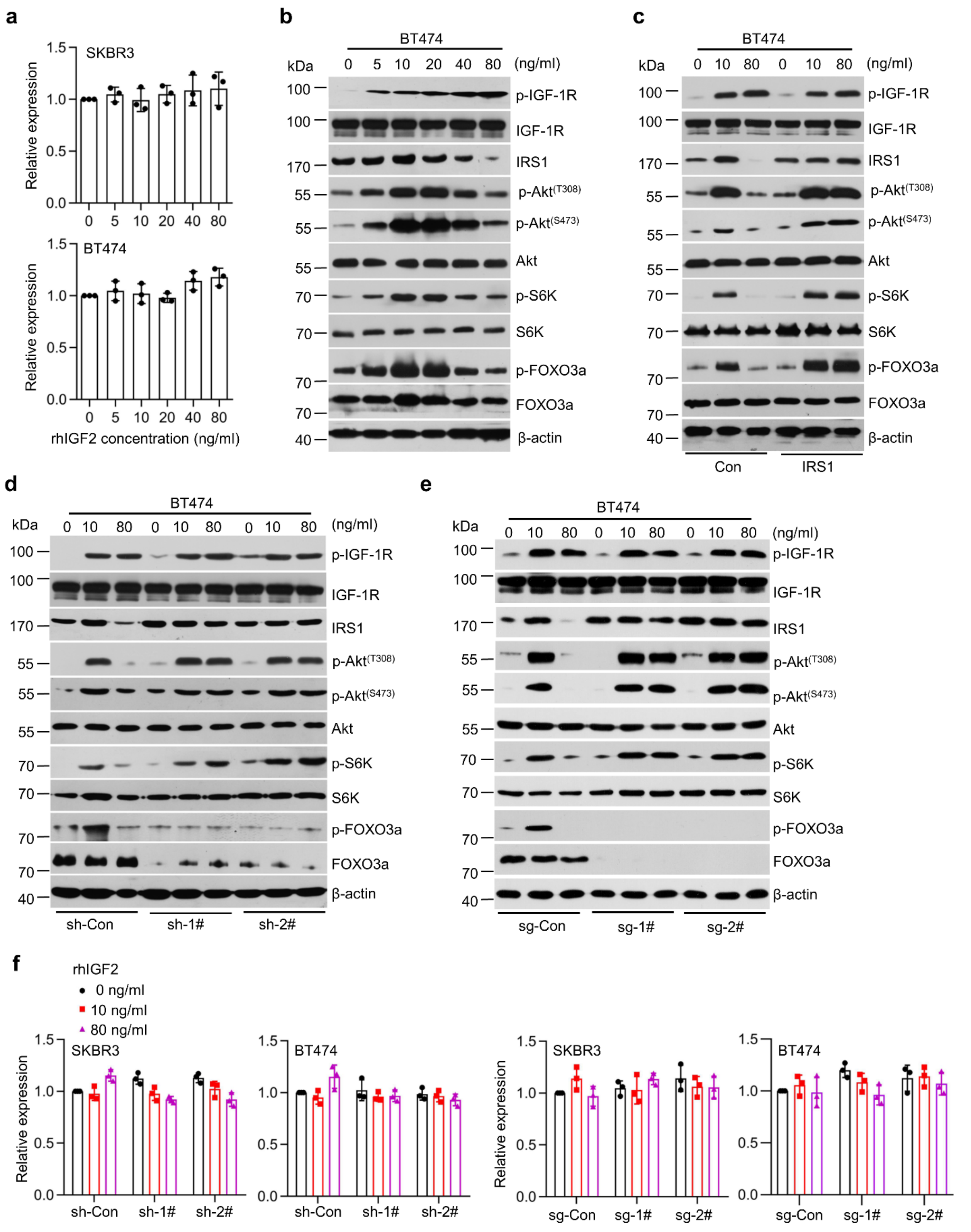

Supplementary figure 2. A negative feedback inhibition loop of the IGF2/IGF-1R/IRS1/mTOR signaling was observed in HER2-positive breast cancer cells.

a SKBR3 or BT474 cells were treated with rhIGF2 at indicated concentrations for 24 hrs. The expression of *IRS1* mRNA was measured by qRT-PCR. **b** BT474 cells were treated with rhIGF2 at indicated concentrations for 24 hrs. The expression of p-IGF-1R, IGF-IR, IRS1, p-Akt$^{(S473)}$, p-

Akt$^{(T308)}$, Akt, p-S6K, S6K, p-FOXO3a, FOXO3a, and β-actin was examined by western blot assays. **c** BT474 cells were transfected with a control vector (Con) or the same vector containing an *IRS1* cDNA (IRS1), followed by rhIGF2 treatment at indicated concentrations for 24 hrs. The cells were examined by western blot assays. **d-f** The indicated cells with downregulation of FOXO3a by either specific shRNAs **(d)** or *FOXO3a* gene deletion via CRISPER-Cas9 technology **(e)** were treated with rhIGF2 at indicated concentrations for 24 hrs. (**d, e**) The cells were examined by western blot assays. The mRNA expression levels of *IRS1* was measured by qRT-PCR **(f)**. n = 3 biologically independent samples (a, f). Data are presented as mean values ± SEM (a, f). Data show a representative of three independent experiments (b, c, d, e). Source data are provided as a Source Data file.

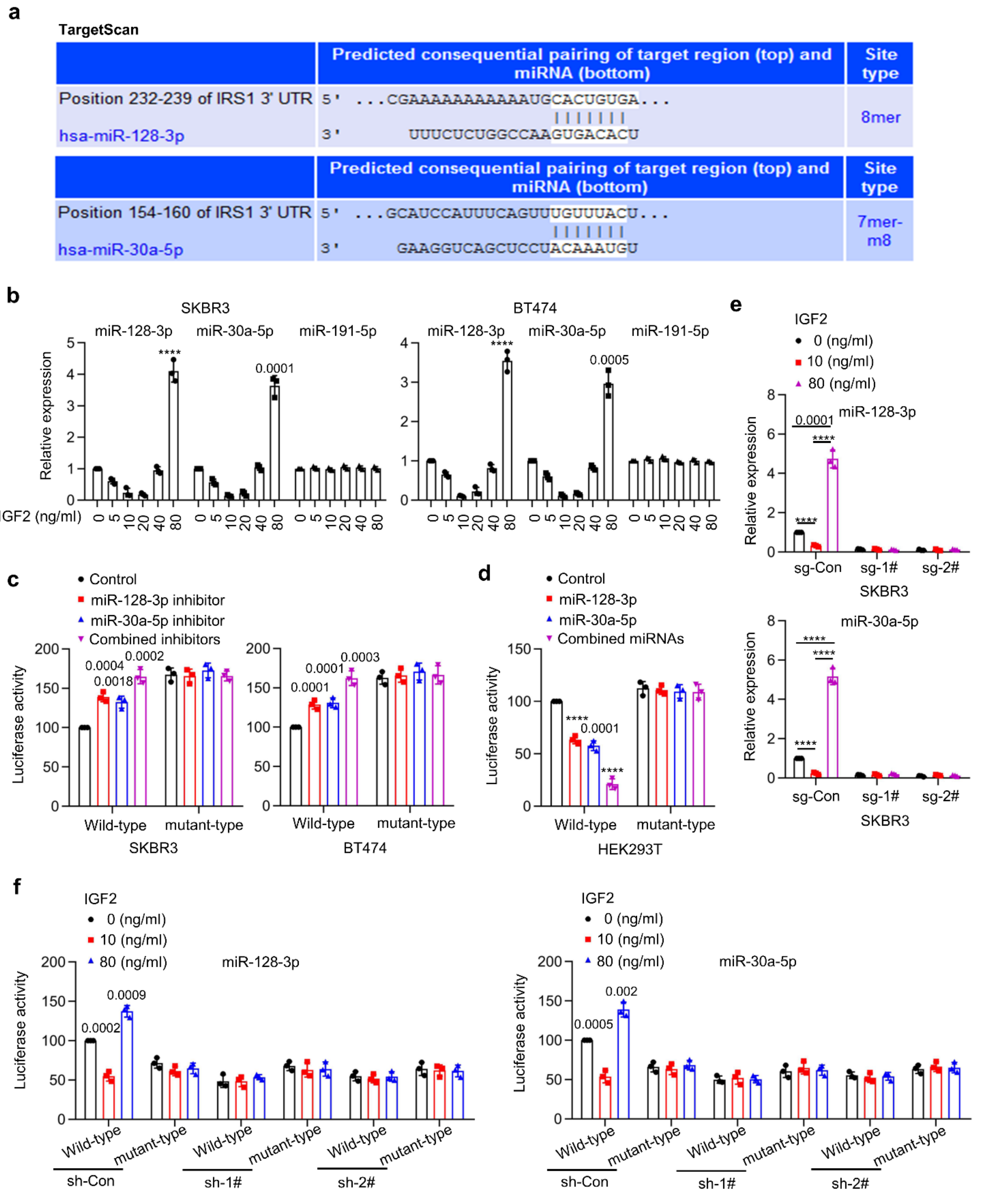

Supplementary figure 3. FOXO3a regulated IRS1 expression via specific miRNAs.

a A schematic representation of the seed sequences of miR-128-3p and miR-30a-5p in the 3'-UTR of *IRS1* mRNA. **b** SKBR3 or BT474 cells were treated with rhIGF2 at indicated concentrations for 24 hrs. The expression levels of miR-128-3p, miR-30-5p, and miR-191-5p were measured by qRT-PCR, **** $p<0.0001$. **c, d** SKBR3 or BT474 cells were co-transfected with a luciferase reporter

vector containing either wild-type *IRS1* 3'-UTR or mutant *IRS1* 3'-UTR along with the inhibitor(s) of miR-128-3p or/and miR-30-5p **(c),** **** $p<0.0001$. HEK293T cells were co-transfected with a luciferase reporter vector containing either wild-type *IRS1* 3'-UTR or mutant *IRS1* 3'-UTR along with the mimic(s) of miR-128-3p or/and miR-30-5p **(d)**. The luciferase activity was determined using the Dual-Luciferase Reporter assays. The Renilla luciferase activity was used as internal control and the firefly luciferase activity was calculated as the mean ± SD after being normalized by Renilla luciferase activity, **** $p<0.0001$. **e** SKBR3 cells with *FOXO3a* gene deletion via CRISPR-Cas9 technology were treated with rhIGF2 at indicated concentrations for 24 hrs. The expression levels of miR-128-3p and miR-30-5p were measured by qRT-PCR, **** $p<0.0001$. **f** SKBR3 cells were transfected with miR-128 or miR-30a promoter reporter vector containing either wild-type or mutant FOXO3a binding sites. The cells were then transfected with control shRNA (sh-Con) or specific *FOXO3a* shRNAs (sh-1# and sh-2#) and treated with rhIGF2 at indicated concentrations. The luciferase activity was measured by the reporter assays. n = 3 biologically independent samples (b-f). Data are presented as mean values ± SEM (b-c, e-f). Statistical significance was determined by a two-tailed Student's t test (b-f). Source data are provided as a Source Data file.

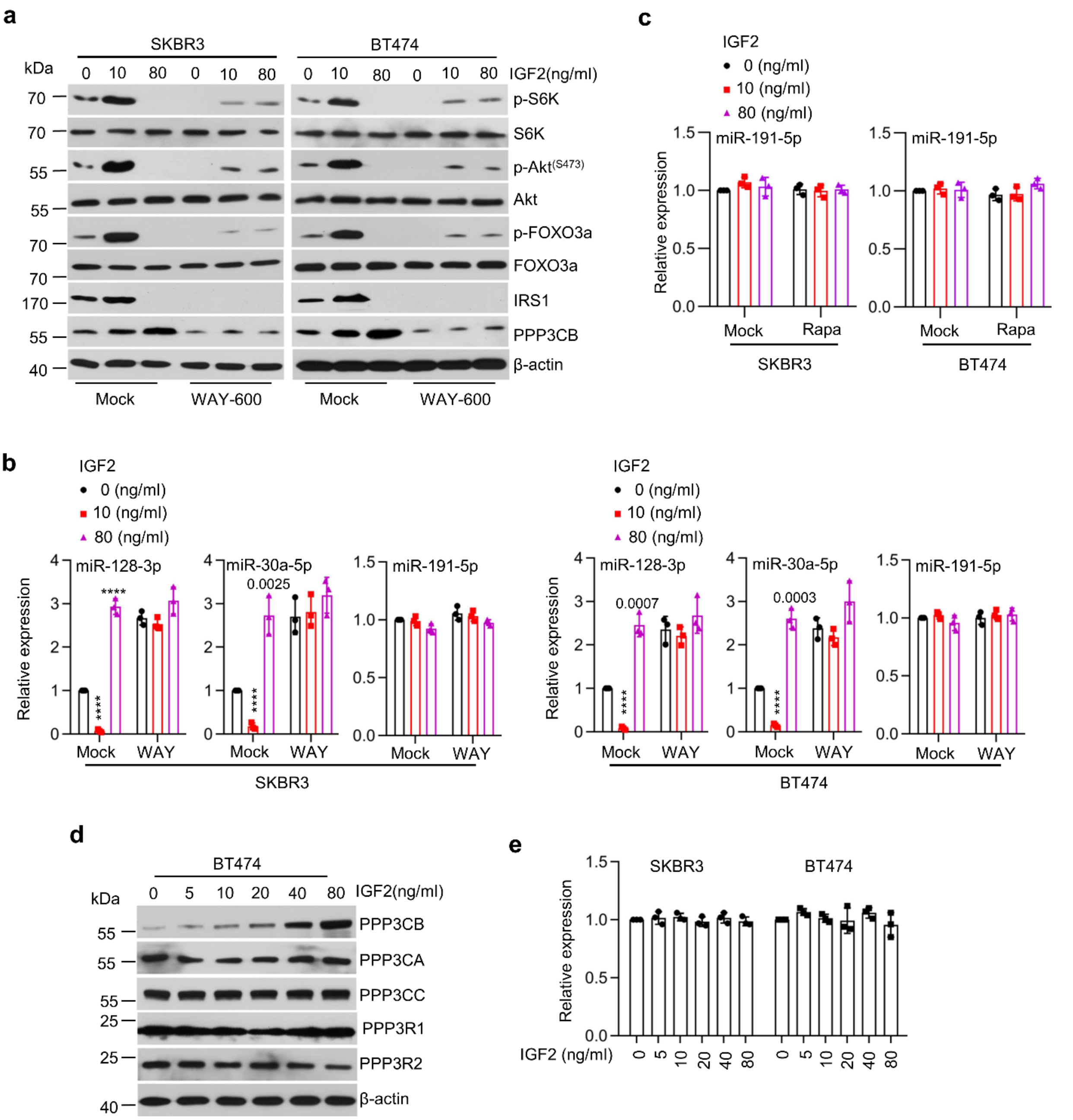

Supplementary figure 4. PPP3CB-mediated reduction of p-FOXO3a controlled the feedback regulation.

a, b SKBR3 or BT474 cells were treated with vehicle (Mock) or mTOR inhibitor WAY-600 (1 μM) in combination with rhIGF2 at indicated concentrations for 24 hrs. The expression levels of p-Akt$^{(S473)}$, Akt, p-S6K, S6K, p-FOXO3a, FOXO3a, IRS1, PPP3CB, and β-actin was examined by western blot assays **(a)**. The expression levels of miR-128-3p, miR-30a-5p, and miR-191-5p were measured by qRT-PCR **(b)**, **** $p<0.0001$. **c** SKBR3 or BT474 cells were treated with vehicle (Mock) or Rapamycin (10 μM, Rapa) in combination with rhIGF2 at indicated concentrations for 24

hrs. The expression level of miR-191-5p was measured by qRT-PCR. **d** BT474 cells were treated with rhIGF2 at indicated concentrations for 24 hrs. The expression of PPP3CB, PPP3CA, PPP3CC, PPP3R1, PPP3R2, and β-actin was examined by western blot assays. **e** SKBR3 or BT474 cells were treated with rhIGF2 at indicated concentrations for 24 hrs. The expression of *PPP3CB* was measured by qRT-PCR. n = 3 biologically independent samples (b, d). Data are presented as mean values ± SEM (b, d). Statistical significance was determined by a two-tailed Student's t test (b, d). Data show a representative of three independent experiments (a, c). Source data are provided as a Source Data file.

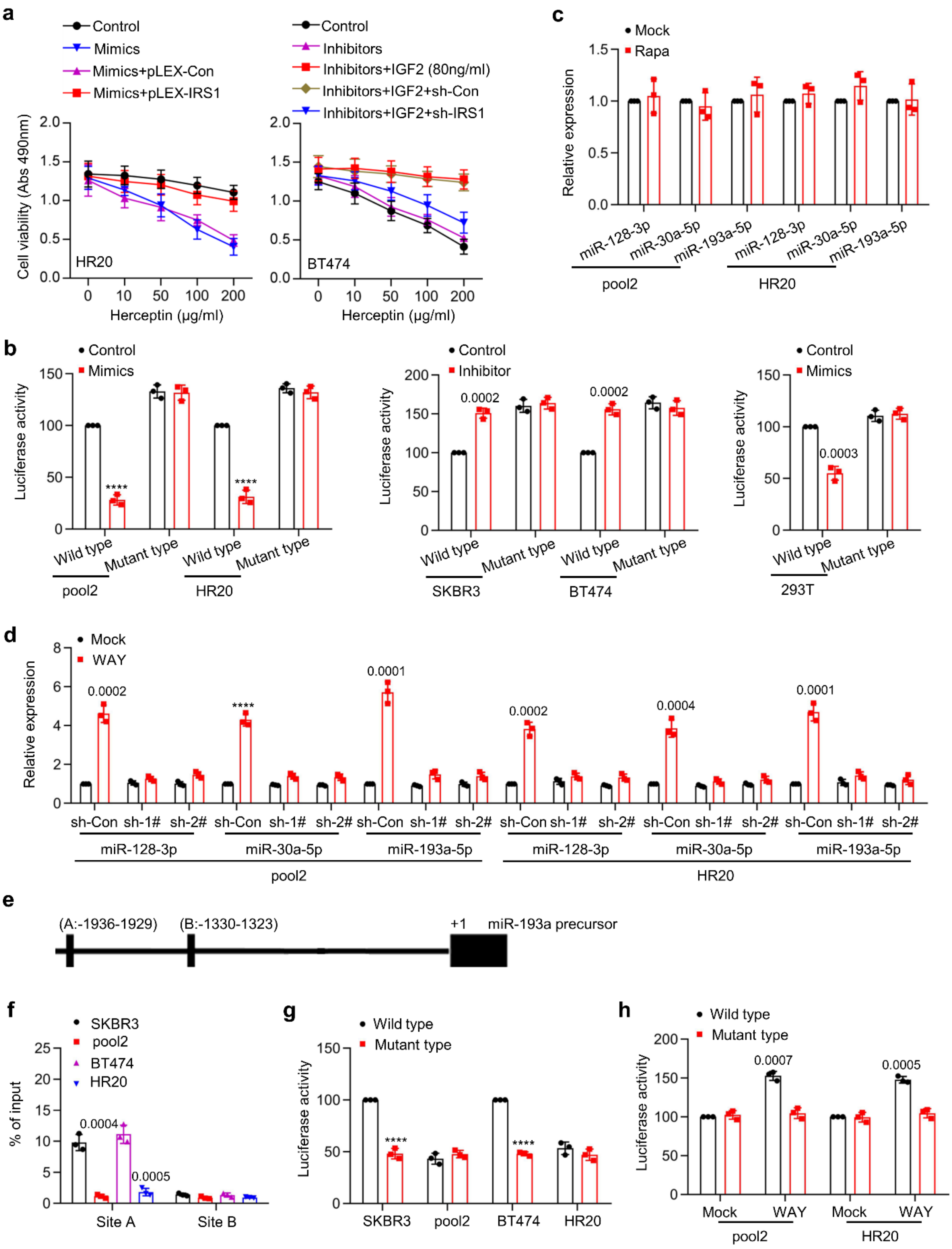

Supplementary figure 5. Dysregulation of FOXO3a-miRNAs axis conferred Herceptin resistance.

a HR20 cells were transfected with miRNA mimics in combination with IRS1 expression vector (pLEX-IRS1), and then treated with Herceptin for 72 hrs. BT474 cells were transfected miRNA

inhibitors in combination with *IRS1* shRNA (sh-IRS1), and then treated with rhIGF2. The cells' sensitivity to Herceptin was evaluated by MTS assays. **b** The indicated cells were co-transfected with a luciferase reporter containing wild type or mutant *IGF2* 3'-UTR along with miR-193a-5p mimics or inhibitor. The luciferase activity was determined by the Dual-Luciferase Reporter assays. The renilla luciferase activity was used as internal control, **** $p<0.0001$. **c** Pool2 or HR20 cells were treated with vehicle (Mock) or Rapamycin (Rapa). The levels of miR-128-3p, miR-30a-5p, and miR-193a-5p were measured by qRT-PCR. **d** Pool2 or HR20 cells were transfected with a control shRNA (sh-Con) or *FOXO3a* shRNA (sh-1# or sh-2#) followed by treatment with vehicle (Mock) or WAY-600 (WAY). The levels of miR-128-3p, miR-30a-5p, and miR-193a-5p were measured by qRT-PCR, **** $p<0.0001$. **e** A schematic representation of FOXO3a binding sites within the 2kb putative promoter region of miR-193a. The first base of the precursor of miR-193a is defined as '+1'. **f** ChIP-qPCR assays were conducted to validate the binding of FOXO3a at miR-193a promoter. **g** miR-193a promoter containing predicted FOXO3a binding sites was cloned into the pGL4 vector. The luciferase activities in the cells were determined by the Dual-Luciferase Reporter assays, **** $p<0.0001$. **h** Pool2 or HR20 cells were transfected with wild type or mutant miR-193a promoter reporter followed by treatment with vehicle (Mock) or WAY-600 (WAY). The luciferase activities were examined by the Dual-Luciferase Reporter assays. n = 3 biologically independent samples (a, b, c, d, f-h). Data are presented as mean values ± SEM (a, c, d, f-h). Statistical significance was determined by a two-tailed Student's t test (b, d, f-h). Source data are provided as a Source Data file.

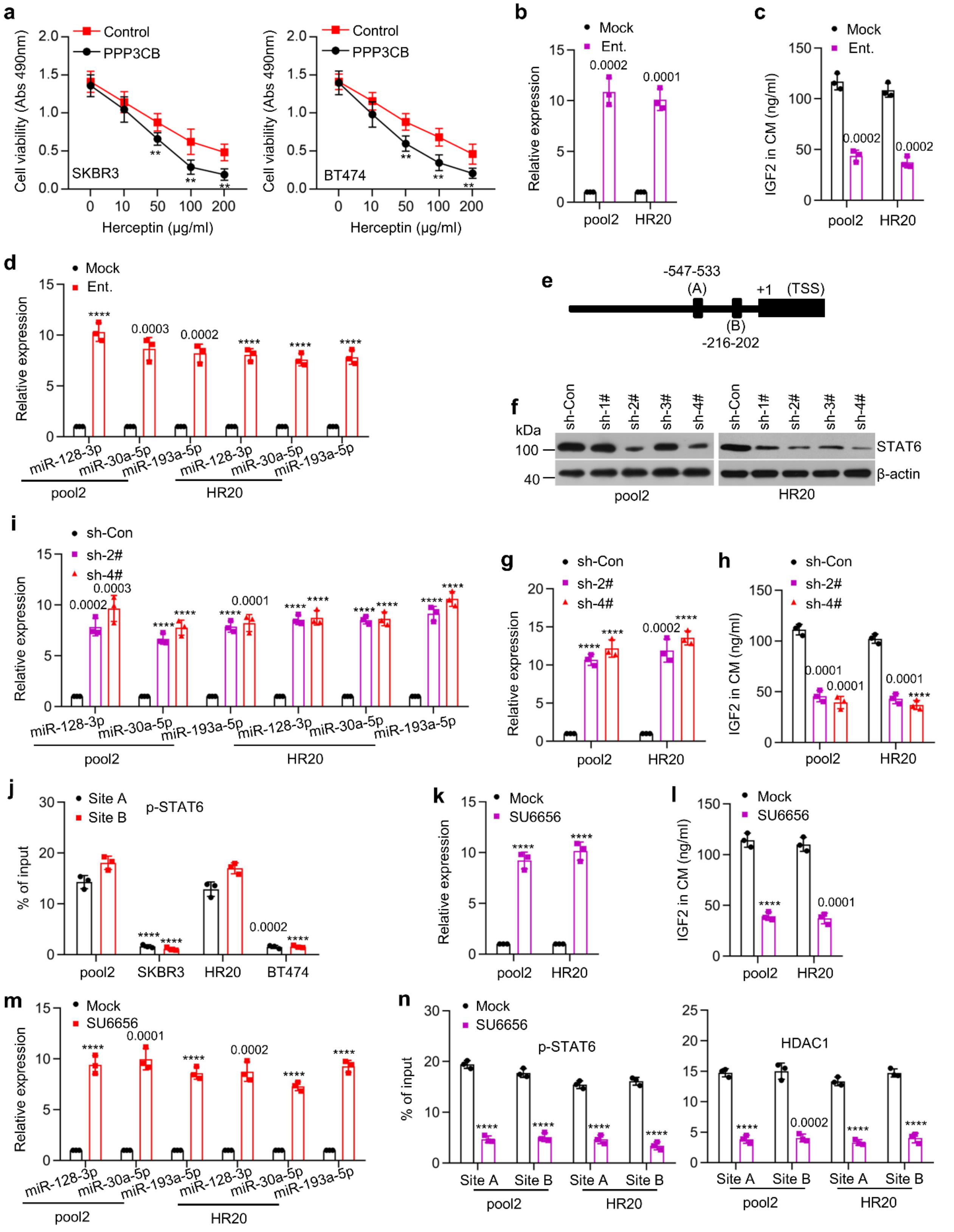

Supplementary figure 6. Src-mediated p-STAT6/HDAC1 complex decreased PPP3CB expression in Herceptin-resistant cells.

a SKBR3 or BT474 cells transfected with control vector (Control) or PPP3CB expression vector (PPP3CB) were treated with Herceptin for 72 hrs. Cell viability was evaluated by MTS assays,

SKBR3 PPP3CB: ** *p*=0.0082, ** *p*=0.0045, ** *p*=0.0011, BT474 PPP3CB: ** *p*=0.0026, ** *p*=0.0013, ** *p*=0.005. **b-d** Pool2 or HR20 cells were treated with vehicle (Mock) or entinostat (1 μM, Ent.) for 24 hrs. The expression of *PPP3CB* mRNA was detected by qRT-PCR **(b)**. IGF2 levels in the CM were examined by ELISA **(c)**. The levels of miR-128-3p, miR-30a-5p, and miR-193a-5p were measured by qRT-PCR **(d)**, **** *p*<0.0001. **e** A schematic representation of STAT6 binding sites within the 2kb putative promoter region of *PPP3CB* upstream the transcription start site. The first base of the transcription start site is defined as '+1'. **f-i** Pool2 or HR20 cells were transfected with control shRNA (sh-Con) or specific *STAT6* shRNAs (sh-1#, sh-2#, sh-3#, or sh-4#). The expression of STAT6 and β-actin was examined by western blots **(f)**. The expression of *PPP3CB* mRNA was detected by qRT-PCR **(g)**, **** *p*<0.0001. IGF2 levels in the CM were examined by ELISA **(h)**, **** *p*<0.0001. The levels of miR-128-3p, miR-30a-5p, and miR-193a-5p were measured by qRT-PCR **(i)**, **** *p*<0.0001. **j** ChIP-qPCR assays were conducted to validate the binding of p-STAT6 at the promoter region of *PPP3CB*, **** *p*<0.0001. **k-m** Pool2 or HR20 cells were treated with vehicle (Mock) or SU6656. The expression of *PPP3CB* mRNA was detected by qRT-PCR **(k)**, **** *p*<0.0001. IGF2 levels in the CM were examined by ELISA **(l)**, **** *p*<0.0001. The levels of miR-128-3p, miR-30a-5p, and miR-193a-5p were measured by qRT-PCR **(m)**, **** *p*<0.0001. The enrichment of p-STAT6 or HDAC1 at *PPP3CB* promoter was determined by ChIP-qPCR **(n)**, **** *p*<0.0001. n = 3 biologically independent samples (a-d, g-l). Data are presented as mean values ± SEM (a-d, g-l). Statistical significance was determined by a two-tailed Student's t test (a-d, g-l). Source data are provided as a Source Data file.

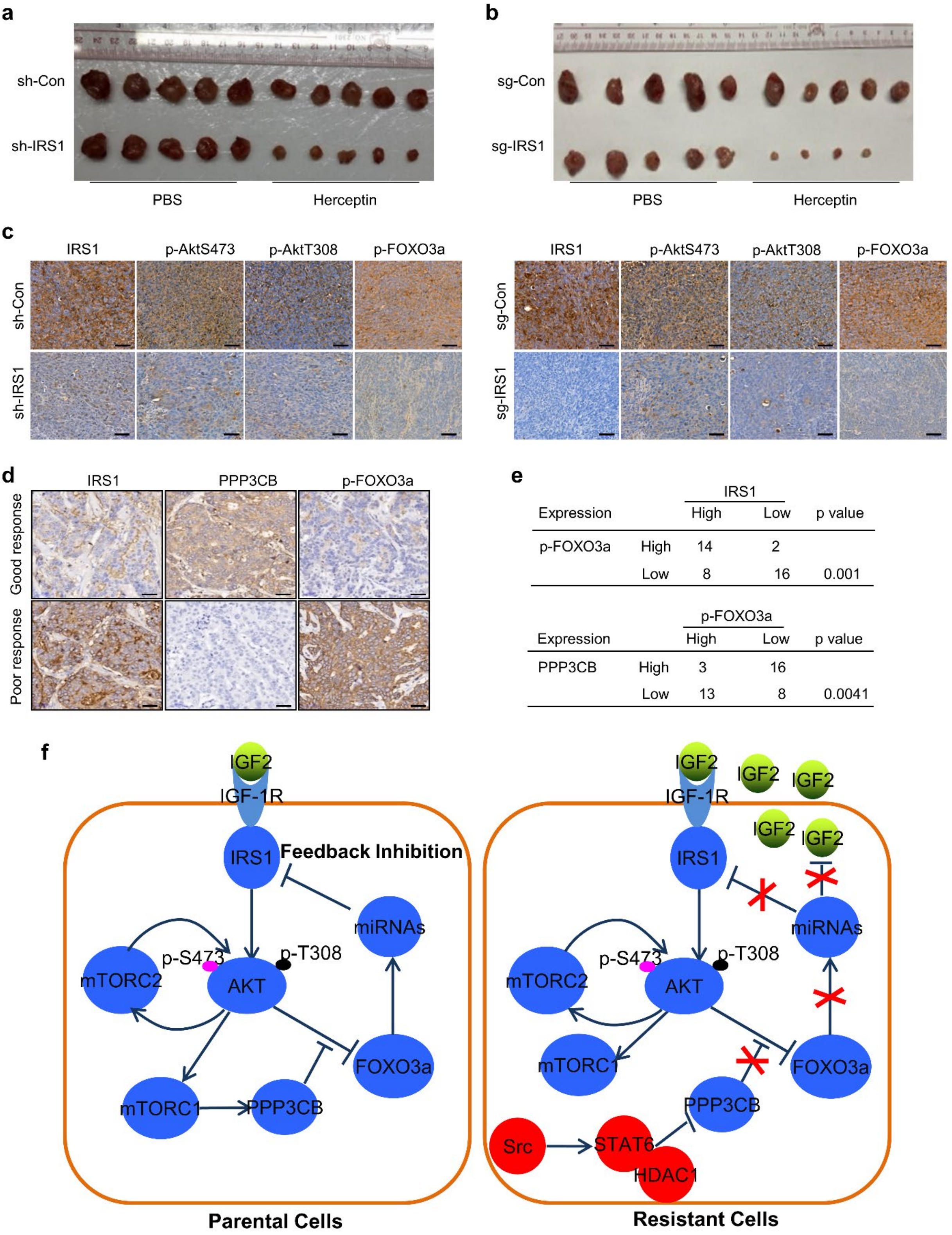

Supplementary figure 7. PPP3CB/FOXO3a/IRS1 signaling contributed to the poor response to Herceptin-containing treatments in HER2-positive breast cancer patients.

a Pool2/sh-Con and pool2/sh-IRS1 cells were subcutaneously inoculated into the armpit of female Balb/C athymic nude mice to generate xenograft tumors. When tumors reached ~100 mm^3, the mice were randomly grouped and received i.p. injection of Herceptin (10 mg/kg) or PBS (n=5) once every

5 days. At the experimental endpoint, the tumors were excised and photographed. **b** Pool2 cells with control sgRNA (sg-Con) or *IRS1* gene specific sgRNA (sg-IRS1) were subcutaneously inoculated into the armpit of female Balb/C athymic nude mice to generate xenograft tumors. When tumors reached ~100 mm^3, the mice were randomly grouped and received i.p. injection of Herceptin (10 mg/kg) or PBS (n=5) once every 5 days. At the experimental endpoint, the tumors were excised and photographed. **c** The expression of IRS1, p-Akt^{S473}, p-Akt^{T308} and p-FOXO3a in the tumors obtained from the animal experiments were examined by IHC assays. Scale bar = 50 µm. **d** The expression of IRS1, PPP3CB, and p-FOXO3a in representative cancer specimens was examined by IHC assays. Scale bar = 50 µm. **e** The correlation between the expression levels of IRS1 and p-FOXO3a or PPP3CB and p-FOXO3a was analyzed. **f** A proposed model of the PPP3CB/FOXO3a/IRS1 signaling dysregulation conferring Herceptin resistance. Data show a representative of three independent experiments (c, d).

Supplementary Table 1. Primers for RT-qPCR analyses of gene expression

Primer name	Sequence (5' to 3')
miR-128-3p-F	CGTCACAGTGAACCGGTCTCTTT
miR-30a-5p-F	CGCTGTAAACATCCTCGACTGGAAG
miR-193a-5p-F	TATGGGTCTTTGCGGGCGA
miR-191-5p-F	CAACGGAATCCCAAAAGCAGCTG
RNU6 and Universal Reverse primers	Purchased from GeneCopoeia Inc (http://www.fulengen.com/) Guangzhou, China.
IGF1-F	GCTCTTCAGTTCGTGTGTGGA
IGF1-R	GCCTCCTTAGATCACAGCTCC
IGF2-F	GTGGCATCGTTGAGGAGTG
IGF2-R	CACGTCCCTCTCGGACTTG
IRS1-F	CTGCACAACCGTGCTAAGG
IRS1-R	CGTCACCGTAGCTCAAGTCC
PPP3CB-F	CCCCAACACATCGCTTGACAT
PPP3CB-R	GGCAGCACCCTCATTGATAATTC
GAPDH-F	AAGGTGAAGGTCGGAGTCAA
GAPDH-R	AATGAAGGGGTCATTGATGG

Supplementary Table 2. Primers for ChIP-qPCR analyses:

Primer name	Sequence (5' to 3')
miR-128a - Site A-F	TGATAGTTTATTGTTTTTGAGTTTT
miR-128a - Site A-R	CAGATTGGGATAGTAGAATGTAGAG
miR-128a - Site B-F	TCAAACAACACTTGCTTACCC
miR-128a - Site B-R	ACTTTTAAACATTACTTGTGCCAC
miR-128a - Site C-F	CAGGACTAACATTACTGCTGGGGA
miR-128a - Site C-R	GAAAGTCTTATGTGTGTAGCCAGGG
miR-30a - Site A-F	TAAAAGTGGTAAGATTCCAAGGCA
miR-30a - Site A-R	TAGATAAATACCAAGGCTTACACCC
miR-30a - Site B-F	TTTTCACCTGCTGACTCAAAGATTA
miR-30a - Site B-R	CAAAAATAAAATCCACACAAAAAGC
miR-30a - Site C-F	CTTTCACTAGATTCAGAATGGTTTT
miR-30a - Site C -R	ACACAGAACCTATCACTCACAACAG
miR-193a - Site A-F	TGTGCCACCACGCCCAGCTAAT
miR-193a - Site A -R	CGCACACCTGTAATCCCAGCACTTT
miR-193a - Site B-F	TCCCACTCCTTTTCCACAGTCTCC
miR-193a - Site B-R	GTACCGGGCAGCATACTTTCTTGAG
PPP3CB - Site A-F	CCATAAGTCACCTGGGCCCAGA
PPP3CB - Site A-R	GTCTGAAAGCACAGAATAAGACTGC
PPP3CB - Site B-F	CTCTGGTCCCCTACCAACCCTTC
PPP3CB - Site B-R	GCATGCAGAATGAAGAATGAGGAGG
