# Supplementary material for: Disruption of FOXO3a-miRNA feedback inhibition of IGF2/IGF-1R/IRS1 signaling confers Herceptin resistance in HER2-positive breast cancer
Source: Nat Commun. 2021 May 11;12:2699. doi: 10.1038/s41467-021-23052-9 (PMC8113606; doi:10.1038/s41467-021-23052-9)
Supplement: Supplementary file 3 — Reporting Summary [file 41467_2021_23052_MOESM3_ESM.pdf]

nature research

Corresponding author(s): Zhimin He, Bolin Lin, Guopei Zheng

Last updated by author(s): Mar 27, 2021

Reporting Summary

Nature Research wishes to improve the reproducibility of the work that we publish. This form provides structure for consistency and transparency in reporting. For further information on Nature Research policies, see our Editorial Policies and the Editorial Policy Checklist.

Statistics

For all statistical analyses, confirm that the following items are present in the figure legend, table legend, main text, or Methods section.

n/a	Confirmed	
☐	☒	The exact sample size (*n*) for each experimental group/condition, given as a discrete number and unit of measurement
☐	☒	A statement on whether measurements were taken from distinct samples or whether the same sample was measured repeatedly
☐	☒	The statistical test(s) used AND whether they are one- or two-sided<br>*Only common tests should be described solely by name; describe more complex techniques in the Methods section.*
☒	☐	A description of all covariates tested
☒	☐	A description of any assumptions or corrections, such as tests of normality and adjustment for multiple comparisons
☐	☒	A full description of the statistical parameters including central tendency (e.g. means) or other basic estimates (e.g. regression coefficient) AND variation (e.g. standard deviation) or associated estimates of uncertainty (e.g. confidence intervals)
☐	☒	For null hypothesis testing, the test statistic (e.g. *F*, *t*, *r*) with confidence intervals, effect sizes, degrees of freedom and *P* value noted<br>*Give P values as exact values whenever suitable.*
☒	☐	For Bayesian analysis, information on the choice of priors and Markov chain Monte Carlo settings
☒	☐	For hierarchical and complex designs, identification of the appropriate level for tests and full reporting of outcomes
☒	☐	Estimates of effect sizes (e.g. Cohen's *d*, Pearson's *r*), indicating how they were calculated

Our web collection on statistics for biologists contains articles on many of the points above.

Software and code

Policy information about availability of computer code

Data collection	CFX96 Real-Time PCR System operated by Bio-Rad iQ5 program; 3DHISTECH; BioTeK
Data analysis	Excel 2013; Prism7 Graphpad (v7.0); ImageJ software (v2.0.0);

For manuscripts utilizing custom algorithms or software that are central to the research but not yet described in published literature, software must be made available to editors and reviewers. We strongly encourage code deposition in a community repository (e.g. GitHub). See the Nature Research guidelines for submitting code & software for further information.

Data

Policy information about availability of data

All manuscripts must include a data availability statement. This statement should provide the following information, where applicable:

- Accession codes, unique identifiers, or web links for publicly available datasets
- A list of figures that have associated raw data
- A description of any restrictions on data availability

All the summary or representative data generated and supporting the findings of this study are available within the paper. All primary data are available from the corresponding author upon reasonable request.

Field-specific reporting

Please select the one below that is the best fit for your research. If you are not sure, read the appropriate sections before making your selection.

☒ Life sciences ☐ Behavioural & social sciences ☐ Ecological, evolutionary & environmental sciences

For a reference copy of the document with all sections, see nature.com/documents/nr-reporting-summary-flat.pdf

Life sciences study design

All studies must disclose on these points even when the disclosure is negative.

Sample size	For in vitro studies, a sample size of n=3 would allow for adequate analysis to reach meaningful conclusions of the data. For in vivo studies, a bigger sample size (n=5) was used to compensate for the higher natural variance in vivo.
Data exclusions	No data was excluded.
Replication	Data show a representative of three independent experiments. All the experimental findings were reliably reproduced.
Randomization	All mice were randomly allocated into experimental groups. For human tissue samples, the group design was conducted in patients who responded to Herceptin-containing treatments. All cells used throughout the study were differentially treated and analyzed in parallel to minimize experimental variation. Samples and corresponding controls were processed at the same time and there were no significant differences between groups at the time of intervention.
Blinding	The experiments were not blinded due to feasibility. Samples were processed through standard procedures.

Reporting for specific materials, systems and methods

We require information from authors about some types of materials, experimental systems and methods used in many studies. Here, indicate whether each material, system or method listed is relevant to your study. If you are not sure if a list item applies to your research, read the appropriate section before selecting a response.

Materials & experimental systems

n/a	Involved in the study	
☐	☒	Antibodies
☐	☒	Eukaryotic cell lines
☒	☐	Palaeontology and archaeology
☐	☒	Animals and other organisms
☐	☒	Human research participants
☒	☐	Clinical data
☒	☐	Dual use research of concern

Methods

n/a	Involved in the study	
☒	☐	ChIP-seq
☒	☐	Flow cytometry
☒	☐	MRI-based neuroimaging

Antibodies

Antibodies used	Rabbit anti-IRS-1 Antibody (Cell Signaling Technology, Cat. #2382, 1:1000 for western blot), Rabbit anti-IGF-I Receptor β (Cell Signaling Technology, Cat. #3018, 1:1000 for western blot), Rabbit anti-Phospho-IGF-I Receptor β (Tyr1131)/Insulin Receptor β (Tyr1146) (Cell Signaling Technology, Cat. #80732, 1:1000 for western blot), Rabbit anti-Akt (Cell Signaling Technology, Cat. #4691, 1:1000 for western blot), Rabbit anti-Phospho-Akt (Thr308) (Cell Signaling Technology, Cat. #13038, 1:1000 for western blot), Rabbit anti-Phospho-Akt (Ser473) (Cell Signaling Technology, Cat. #4060, 1:1000 for western blot, 1:100 for immunohistochemistry), Rabbit anti-p70 S6 Kinase (Cell Signaling Technology, Cat. #2708, 1:1000 for western blot), Rabbit anti-Phospho-p70 S6 Kinase (Thr389) (Cell Signaling Technology, Cat. #97596, 1:1000 for western blot), Rabbit anti-FOXO3a (Cell Signaling Technology, Cat. #12829, 1:1000 for western blot), Rabbit anti-Phospho-FOXO3a (Ser253) (Cell Signaling Technology, Cat. #9466, 1:1000 for western blot), Rabbit anti-STAT6 (Cell Signaling Technology, Cat. #5397, 1:1000 for western blot), Rabbit anti-Phospho-Stat6 (Tyr641) (Cell Signaling Technology, Cat. #56554, 1:1000 for western blot, 1:100 for Chromatin immunoprecipitation), Rabbit anti-Src (Cell Signaling Technology, Cat. #2109, 1:1000 for western blot), Rabbit anti-Phospho-Src Family (Tyr416) (Cell Signaling Technology, Cat. #59548, 1:1000 for western blot), and Rabbit anti-HDAC1 (Cell Signaling Technology, Cat. #34589, 1:1000 for western blot, 1:50 for Chromatin immunoprecipitation), Rabbit anti-PPP3CB (Sigma, Cat. #HPA008823, 1:1000 for western blot, 1:50 for immunohistochemistry), Mouse anti-PPP3CA (Sigma, Cat. #WH0005530M3, 1:1000 for western blot), Mouse anti-PPP3CC (Sigma, Cat. #SAB1409493, 1:1000 for western blot), Mouse anti-PPP3R1 (Sigma, Cat. #WH0005534M1, 1:1000 for western blot), Mouse anti-PPP3R2 (Sigma, Cat. #SAB1406289, 1:1000 for western blot), Mouse anti-β-actin (Sigma, Cat. #A5316, 1:3000 for western blot), Rabbit anti-FOXO3a (Thermo Scientific, Cat. #720128, 1:50 for Chromatin immunoprecipitation), Mouse anti-IRS-1 Antibody (Thermo Scientific, Cat. #MA5-36222, 1:50 for immunohistochemistry), Rabbit anti-Phospho-Akt (Thr308) (Thermo Scientific, Cat. #44-602G, 1:100 for immunohistochemistry), Rabbit anti-Phospho-FOXO3a (Ser253) (Thermo Scientific, Cat. #PA5-36816, 1:50 for immunohistochemistry), Goat anti-Rabbit IgG (H+L) Secondary Antibody, HRP (Thermo Scientific, Cat. #31460, 1:5000 for western blot), Goat anti-Mouse IgG (H+L) Secondary Antibody, HRP (Thermo Scientific, Cat. #31430, 1:5000 for western blot).

Validation

All antibodies have been validated on the product webpages or literature, including:
1. anti-IRS-1 antibody has been validated for WB of human samples by previous publications (e.g .PMID: 32248666,PMID: 32576196, and PMID: 32660483). https://www.cellsignal.com/products/primary-antibodies/irs-1-antibody/2382?site-search-type=Products&N=4294956287&Ntt=+anti-irs-1&fromPage=plp
2. anti-IGF-I Receptor β antibody has been validated for WB of human samples by previous publications (e.g .PMID: 31073032 and PMID: 30993883). https://www.cellsignal.com/products/primary-antibodies/igf-i-receptor-b-111a9-rabbit-mab/3018?site-search-type=Products&N=4294956287&Ntt=anti-igf-i+receptor+%CE%B2+&fromPage=plp
3. anti-Phospho-IGF-I Receptor β (Tyr1131)/Insulin Receptor β (Tyr1146) antibody has been validated for WB of human samples by previous publications (e.g .PMID: 31354352). https://www.cellsignal.com/products/primary-antibodies/phospho-igf-i-receptor-b-tyr1131-insulin-receptor-b-tyr1146-d6d5l-rabbit-mab/80732?site-search-type=Products&N=4294956287&Ntt=anti-phospho-igf-i+receptor+%CE%B2+%28tyr1131%29%2Finsulin+receptor+%CE%B2+%28tyr1146%29&fromPage=plp
4. anti-Akt antibody has been validated for WB of human samples by previous publications (e.g .PMID: 33268783 and PMID: 33080033). https://www.cellsignal.com/products/primary-antibodies/akt-pan-c67e7-rabbit-mab/4691?site-search-type=Products&N=4294956287&Ntt=anti-akt&fromPage=plp
5. anti-Phospho-Akt (Thr308) antibody has been validated for WB of human samples by previous publications (e.g .PMID: 33177525 and PMID: 32901877). https://www.cellsignal.com/products/primary-antibodies/phospho-akt-thr308-d25e6-xp-rabbit-mab/13038?site-search-type=Products&N=4294956287&Ntt=anti-phospho-akt+%28thr308%29&fromPage=plp
6. anti-Phospho-Akt (Ser473) antibody has been validated for WB and IHC of human samples by previous publications (e.g .PMID: 33355374, PMID: 33293521 and PMID: 32792479). https://www.cellsignal.com/products/primary-antibodies/phospho-akt-ser473-d9e-xp-rabbit-mab/4060?site-search-type=Products&N=4294956287&Ntt=anti-phospho-akt+%28ser473%29&fromPage=plp
7. anti-p70 S6 Kinase antibody has been validated for WB of human samples by previous publications (e.g .PMID: 33268783 and PMID: 32968434). https://www.cellsignal.com/products/primary-antibodies/p70-s6-kinase-49d7-rabbit-mab/2708?site-search-type=Products&N=4294956287&Ntt=anti-p70+s6+kinase&fromPage=plp
8. anti-Phospho-p70 S6 Kinase (Thr389) antibody has been validated for WB of human samples by previous publications (e.g .PMID: 31604167 and PMID: 31702028). https://www.cellsignal.com/products/primary-antibodies/phospho-p70-s6-kinase-thr389-d5u1o-rabbit-mab/97596?site-search-type=Products&N=4294956287&Ntt=anti-phospho-p70+s6+kinase+%28thr389%29+&fromPage=plp
9. anti-FOXO3a antibody has been validated for WB of human samples by previous publications (e.g .PMID: 32051395 and PMID: 32210729). https://www.cellsignal.com/products/primary-antibodies/foxo3a-d19a7-rabbit-mab/12829?site-search-type=Products&N=4294956287&Ntt=anti-foxo3a&fromPage=plp
10. anti-Phospho-FOXO3a (Ser253) antibody has been validated for WB of human samples by previous publications (e.g .PMID: 32760250 and PMID: 32424251). https://www.cellsignal.com/products/primary-antibodies/phospho-foxo3a-ser253-antibody/9466?site-search-type=Products&N=4294956287&Ntt=anti-phospho-foxo3a+%28ser253%29&fromPage=plp
11. anti-STAT6 antibody has been validated for WB of human samples by previous publications (e.g .PMID: 32075954 and PMID: 30842418). https://www.cellsignal.com/products/primary-antibodies/stat6-d3h4-rabbit-mab/5397?site-search-type=Products&N=4294956287&Ntt=anti-stat6&fromPage=plp
12. anti-Phospho-Stat6 (Tyr641) antibody has been validated for WB and ChIP of human samples by previous publications (e.g .PMID: 31492901, PMID: 30842418 and PMID: 30662948). https://www.cellsignal.com/products/primary-antibodies/phospho-stat6-tyr641-d8s9y-rabbit-mab/56554?site-search-type=Products&N=4294956287&Ntt=anti-phospho-stat6+%28tyr641%29+&fromPage=plp
13. anti-Src antibody has been validated for WB of human samples by previous publications (e.g .PMID: 33293521, PMID: 32683421 and PMID: 32391018). https://www.cellsignal.com/products/primary-antibodies/src-36d10-rabbit-mab/2109?site-search-type=Products&N=4294956287&Ntt=anti-src&fromPage=plp
14. anti-Phospho-Src Family (Tyr416) antibody has been validated for WB of human samples by previous publications. https://www.cellsignal.com/products/primary-antibodies/phospho-src-family-tyr416-e6g4r-rabbit-mab/59548?site-search-type=Products&N=4294956287&Ntt=anti-phospho-src+family+%28tyr416%29&fromPage=plp
15. anti-HDAC1 antibody has been validated for WB and ChIP of human samples by previous publications (e.g .PMID: 32630570, PMID: 32181805 and PMID: 31123064). https://www.cellsignal.com/products/primary-antibodies/hdac1-d5c6u-xp-rabbit-mab/34589?site-search-type=Products&N=4294956287&Ntt=anti-hdac1&fromPage=plp
16. anti-PPP3CB antibody has been validated for WB and IHC of human samples by previous publications. https://www.sigmaaldrich.com/catalog/product/sigma/hpa008823?lang=zh&region=CN and https://www.sigmaaldrich.com/catalog/product/sigma/hpa008233?lang=zh&region=CN
17. anti-PPP3CA antibody has been validated for WB of human samples by previous publications. https://www.sigmaaldrich.com/catalog/product/sigma/wh0005530m3?lang=zh&region=CN
18. anti-PPP3CC antibody has been validated for WB of human samples by previous publications. https://www.sigmaaldrich.com/catalog/product/sigma/sab1409493?lang=zh&region=CN
19. anti-PPP3R1 antibody has been validated for WB of human samples by previous publications. https://www.sigmaaldrich.com/catalog/product/sigma/wh0005534m1?lang=zh&region=CN
20. anti-PPP3R2 antibody has been validated for WB of human samples by previous publications. https://www.sigmaaldrich.com/catalog/product/sigma/sab1406289?lang=zh&region=CN
21. anti-β-actin antibody has been validated for WB of human samples by previous publications. https://www.sigmaaldrich.com/catalog/product/sigma/a5316?lang=zh&region=CN
22. anti-FOXO3a antibody has been validated for ChIP of human samples by previous publications (e.g .PMID: 30842454). https://www.thermofisher.com/cn/zh/antibody/product/FOXO3A-Antibody-Polyclonal/720128
23. anti-IRS-1 antibody has been validated for IHC of human samples by previous publications. https://www.thermofisher.com/cn/zh/antibody/product/IRS1-Antibody-clone-10I3-Monoclonal/MA5-36222
24. anti-Phospho-Akt (Thr308) antibody has been validated for IHC of human samples by previous publications. https://www.thermofisher.com/cn/zh/antibody/product/Phospho-AKT1-Thr308-Antibody-Polyclonal/44-602G
25. anti-Phospho-FOXO3a (Ser253) antibody has been validated for IHC of human samples by previous publications. https://www.thermofisher.com/cn/zh/antibody/product/Phospho-FOXO3A-Ser253-Antibody-Polyclonal/PA5-36816
26. anti-Rabbit IgG (H+L) Secondary antibody has been validated for WB of human samples by previous publications (e.g .PMID: 23628792 and PMID: 31300643). https://www.thermofisher.com/cn/zh/antibody/product/Goat-anti-Rabbit-IgG-H-L-Secondary-Antibody-Polyclonal/31460
27. anti-Mouse IgG (H+L) Secondary antibody has been validated for IHC of human samples by previous publications (e.g .PMID:

31015432 and PMID: 29985392). https://www.thermofisher.com/cn/zh/antibody/product/Goat-anti-Mouse-IgG-H-L-Secondary-Antibody-Polyclonal/31430

Eukaryotic cell lines

Policy information about cell lines

Cell line source(s)	Human HER2-positive breast cancer cell lines SKBR3 and BT474 were obtained from the American Type Culture Collection (Manassas, VA, USA). Herceptin-resistant sublines pool2 and HR20 were derived from SKBR3 and BT474 cells, respectively.
Authentication	Cells were authenticated using Short Tandem Repeat (STR) analysis with PowerPlex® 18D System from Promega (Madison, WI, USA).
Mycoplasma contamination	Cells were free of mycoplasma contamination, determined by the MycoAlert™ Mycoplasma Detection Kit (Lonza Group Ltd., Basel, Switzerland) every three months.
Commonly misidentified lines (See ICLAC register)	No commonly misidentified cell lines were used in this study

Animals and other organisms

Policy information about studies involving animals; ARRIVE guidelines recommended for reporting animal research

Laboratory animals	Female 4- to 6-week-old Balb/C athymic nude mice were purchased from Guangdong Medical Laboratory Animal Center. Mice were housed under specific-pathogen-free condition and in individually ventilated cages, under the standard room temperature (22°C) and humidity (55%), 12/12 light/dark cycle. All animal procedures were conducted under the approval of the Institutional Animal Care and Use Committee (IACUC) of Guangzhou Medical University.
Wild animals	No wild animals were used in the study.
Field-collected samples	No field-collected samples were used in this study.
Ethics oversight	The animal studies were approved by the Institutional Animal Care and Use Committee (IACUC) of Guangzhou Medical University.

Note that full information on the approval of the study protocol must also be provided in the manuscript.

Human research participants

Policy information about studies involving human research participants

Population characteristics	HER2-positive breast cancer samples were obtained from 40 female breast cancer patients (22-65 years age, mean=40 years) treated with the neoadjuvant Herceptin-containing regimen TAC (Docetaxel, Doxorubicin and Cyclophosphamide) from the Affiliated Cancer Hospital and Institute of Guangzhou Medical University (Guangzhou, China) between 2008 and 2012. Drs. Ni Qiu and Hongsheng Li, who are physician scientists and our collaborators in the current study, assessed the patients' responses. Good response was defined as CR (Complete Response) with disappearance of all target lesions or PR (Partial Response) with at least 30% decrease in the sum of diameters of target lesions after the neoadjuvant therapy including Herceptin. Poor response was defined as SD (Stable Disease) without sufficient shrinkage to quality for PR or PD (Progressive Disease) with at least 20% increase in the sum of diameters of target lesions.
Recruitment	The clinical samples were collected at the Affiliated Cancer Hospital and Institute of Guangzhou Medical University. Patients with HER2-positive breast cancer admitted to the hospital were selected according to the experimental needs. Informed consent were obtained from involved patients. All samples were collected with no bias.
Ethics oversight	All samples were collected with informed consent from the patients and all examining procedures were performed with the approval of the Internal Review and Ethics Boards (IRB) of the Affiliated Cancer Hospital and Institute of Guangzhou Medical University.

Note that full information on the approval of the study protocol must also be provided in the manuscript.
